# Electrochemical Iodination through the In Situ Generation of Iodinating Agents: A Promising Green Approach

**DOI:** 10.3390/molecules28145555

**Published:** 2023-07-20

**Authors:** Letizia Sorti, Fiammetta Vitulano, Elia Cappellini, Fulvio Uggeri, Carlo Francesco Morelli, Guido Sello, Alessandro Minguzzi, Alberto Vertova

**Affiliations:** 1 Laboratory of Applied Electrochemistry, Dipartimento di Chimica, Università degli Studi di Milano, Via Golgi, 19, 20133 Milano, Italy; 2 Bracco SpA, Via Caduti di Marcinelle, 13, 20134 Milano, Italy; 3 Consorzio Interuniversitario Nazionale per la Scienza e Tecnologia dei Materiali—INSTM, Via G. Giusti 9, 50121 Firenze, Italy

**Keywords:** electro-organic synthesis, green chemistry, iodination reactions, 5-hydroxyisophthalic acid iodination, 5-sulfosalicylic acid iodination, electrosynthesis in aqueous solvent

## Abstract

The synthesis of iodinated compounds using cheap, simple, and green strategies is of fundamental importance. Iodination reactions are mainly used to synthesize useful intermediates, especially in the pharmaceutical field, where they are employed for the production of contrast media or of iodinated active pharmaceutical ingredients. Traditional synthetic methods suffer from the use of erosive, toxic, or hazardous reactants. Approaches which involve the use of molecular iodine as an iodinating agent require the addition of an oxidizing agent, which is often difficult to handle. Electrochemistry can offer a valid and green alternative by avoiding the addition of such oxidizing agents, transforming the iodine source in the active species through the use of electrons as the main reactants. Herein, we report the electrochemical iodination with the generation of iodinating species in situ in water by using iodides as the source of iodine atoms. First of all, the electrochemical behavior of iodide and iodine in water on carbonaceous anodes was studied and, after selecting the suitable potential, in situ electrochemical iodination was successfully applied to 5-hydroxyisophthalic acid and 5-sulfosalicylic acid, comparing the iodinating power of I_2_ and iodonium species.

## 1. Introduction

Electrochemistry has already demonstrated its potential in developing remediation techniques for environmental safeguarding [1,2], but recently its application to the green synthesis of chemicals has been explored. Organic electrosynthesis is in fact coherent (when coupled with the choice of non-toxic solvents, renewable starting reagents, and renewable energy sources) with the green chemistry concept, fulfilling several essential criteria needed for the development of environmentally compatible processes [3].

**Figure 1:**
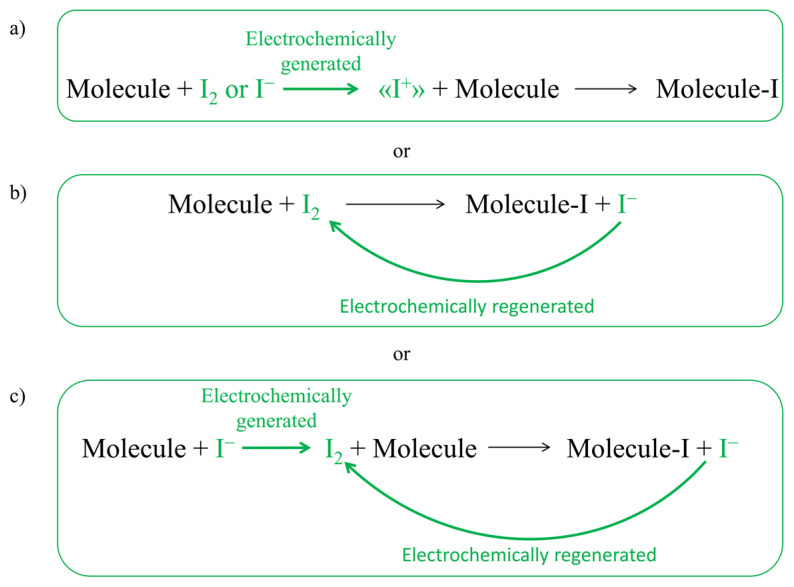
Strategies for the green electrochemical iodination of organic molecules in water. Strategy (**a**) is predicted to be the most reactive one, while strategies (**b**,**c**) are possible only when dealing with highly active organic molecules, able to be iodinated by molecular iodine.

In particular, the research has focused on the development of electrosynthetic routes for common and widely used reactions, such as halogenation reactions. Halogen-containing compounds are useful both by themselves and as highly versatile intermediates in many fields, especially in pharmaceutical synthesis. Among the halogenations, this paper focuses on electrochemical iodination. Currently, the main use of iodination reaction is in the synthesis of intermediates, due to the ability of iodine to act as a leaving group. Some of the most common products of iodination are aromatic iodo compounds for cross-coupling reactions [4,5,6]. In the pharmaceutical industry, iodinated compounds are also widely used as contrast media (iopamidol, iohexol, iodixanol, …) and a rise of interest has been shown for the synthesis of iodinated active pharmaceutical ingredients (APIs). Some common iodinated APIs are povidone-iodine, an antiseptic used for skin disinfection, amiodarone, an antiarrhythmic medication, and triiodothyronine, a thyroid hormone used to treat hypothyroidism. Iodination reactions are currently carried out using toxic and difficult-to-handle reactants, which add steps and costs to the final product, such as ICl [7], N-iodosuccinimide [8], and 1,3-diiodo-5,5-dimethylhydantoin [9,10]. Molecular iodine, which is intrinsically greener, is sometimes used as an iodinating agent, but it has two main drawbacks: low electrophilicity and low atomic efficiency. When dealing with iodination with molecular iodine, in fact, half of iodine atoms are lost as iodides. For this reason, usually its use is coupled with the addition of an oxidizing agent (such as H_2_O_2_) [11]. Electrochemistry can help the reaction by avoiding the addition of such oxidizing agents, transforming the iodine source in the active species using electrons as the main reactants. Electrochemical halogenation has already achieved good results in many cases [12], but it would be particularly useful to find synthetic methods that are even greener, simpler, and therefore more capable of undergoing an industrial scale-up than those investigated so far. To date, in fact, there are still several problems to be solved. For example, the generation of the iodinating agent and the iodination itself are usually carried out separately and in organic solvents, especially acetonitrile, that shows the ability to stabilize the “I^+^” reactive species [12,13,14,15,16,17]. To make the electrochemical iodination as green and simple as possible, in this study, the use of iodides or iodine as a source of iodine atoms for the generation/regeneration of the iodinating species in situ in water was investigated (see Figure 1).

To assess this approach, the electrochemical behavior of iodide and iodine in water on carbonaceous anodes was studied to identify the potential needed to transform them. The first issue when dealing with iodine in water, in fact, is its complex behavior that involves several equilibria and species. Iodonium species I^+^ cannot exist in aqueous systems as such, but it has been postulated that the species HOI, OI^−^, and H_2_OI^+^ can exist, whose lifespan has been investigated within the complex system of hydrolysis in water [18,19,20]. Iodide and iodine electrochemical behavior in water was widely studied by several authors on different electrodes, but there is still no common agreement on the species and the reactions involved [21,22,23,24,25]. Cyclic voltammograms at different pHs of iodide and iodine in water allowed the identification of the reactions and species involved when a GC electrode was used as a working electrode. Then, two molecules, 5-hydroxyisophthalic acid and 5-sulfosalicylic acid, were successfully tested as case studies for the in situ electrochemical iodination, and the iodinating power of I_2_ and iodonium species was compared.

## 2. Results and Discussion

### 2.1. Electrochemical Oxidation of Iodide/Iodine in Aqueous Solvent on Carbonaceous Materials

The first step of this study was focused on understanding which species are involved when the electrochemical oxidation of iodide and iodine is carried out using carbonaceous electrodes. Carbonaceous electrodes were chosen as WE considering their sensitivity to the analytes, their robustness, and relatively low costs. Several carbonaceous materials were tested (glassy carbon, graphite, carbon felt, carbon cloth, and carbon paper) and their similar behavior was verified. Iodide’s and iodine’s similar electrochemical behavior was also verified: iodine, in fact, dissociates in water generating iodide via Equations (1) and (2).

I_2_ + H_2_O ⇆ I^−^ + HIO + H^+^ (1)

3 HIO ⇆ IO_3_^−^ + 2 I^−^ + 3 H^+^ (2)

Given these premises, here the results obtained with a GC as WE and KI as analyte are reported, where GC was chosen for its small capacitance and its reproducibility and KI for its high solubility in water.

**Figure 2:**
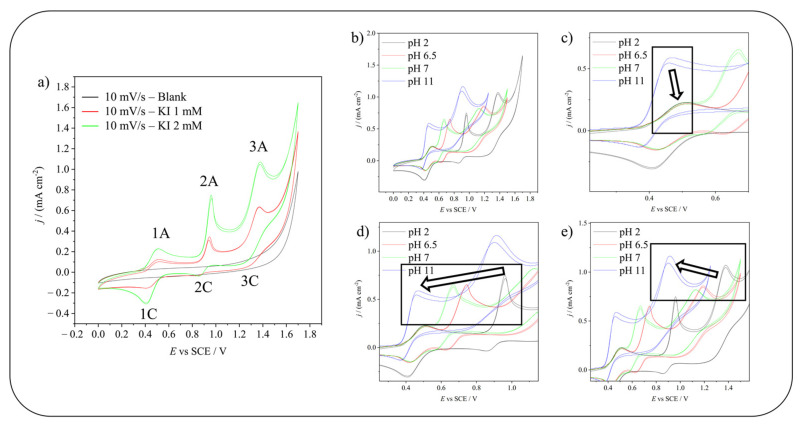
(**a**) CVs on a GC electrode of blank, KI 1 mM, and KI 2 mM in phosphate buffer 0.5 M pH 2. 1A, 2A and 3A are the anodic peaks, while 1C, 2C and 3C are the corresponding cathodic peaks. (**b**) CVs for the pH dependency of peaks position on a GC electrode of KI 2 mM in phosphate buffer 0.5 M solutions. Focus on (**c**) first (1A), (**d**) second (2A), and (**e**) third (3A) anodic peak. IR compensation has been applied.

As shown in Figure 2a, on a GC electrode, KI gives rise to three anodic peaks. By studying the dependency on the pH of the position of these peaks, it is possible to identify the reactions and the species involved. Figure 2b shows the CVs of KI at different pHs and Figure 2c–e focuses on the shift of each peak: first 1A, second 2A, and third 3A, respectively.

The first peak shows no pH dependency and has been identified as the oxidation of iodide to iodine as widely reported in the literature [22,23,24]. The peak shows a shoulder because the reaction probably occurs in two steps, as reported by Miller et al. [22] (see Equations (3) and (4)) and Ito et al. [25] (see Equations (5) and (6)). The shoulder is more visible as the scan rate is increased. The second and the third peaks show pH dependency; the higher the pH, the lower the peak potential (E_peak_ shifts with a dependency on pH at 20 mV s^−1^ of −0.055 pH and −0.047 pH, respectively). At pH 11, the first and the second peaks merge. This pH dependency was used to identify the reactions and species involved in the electrochemical oxidation. By comparing the experimental slope of the line E_peak_ vs. pH of the second and third peak with the theoretical ones of the possible reactions involved, the peaks were identified as reported in Equations (7) and (8) (in Supplementary Materials S1 both the theoretical equations [26] and the experimental data are reported). The equation attributed to 2A fits the one proposed in the literature [22], while the one attributed to the 3A peak may differ from those previously proposed. However, considering that HIO_3_ is thermodynamically stable only at low pH, even the formation of IO_3_^−^ cannot be excluded. Moreover, the possible local pH variation due to the reaction occurring at 2A makes it difficult to uniquely attribute the reaction occurring at peak 3A on the basis of pH dependence alone.
1A2 I^−^ → I_2_ + 2 e^−^E_vs SHE_ (V) = 0.621(3)2 I_3_^−^→ 3 I_2_ + 2 e^−^E_vs SHE_ (V) = 0.789(4)3 I^−^ → I_3_^−^ + 2 e^−^E_vs SHE_ (V) = 0.536(5)2 I^−^ → I_2_ + 2 e^−^E_vs SHE_ (V) = 0.621(6)2AI_2_ + 2 H_2_O → 2 HIO + 2 H^+^ + 2 e^−^E_vs SHE_ (V) = 1.354−0.0591 pH(7)3AI^−^ + 3 H_2_O → HIO_3_ + 5 H^+^ + 6 e^−^E_vs SHE_ (V) = 1.077−0.0493 pH(8)

### 2.2. Electrochemical Iodination of Phenolic Substrates in Aqueous Solvent on Carbonaceous Materials—CVs Monitoring

What was learned from the voltammetric study was then applied to the electrolysis of KI in the presence of a phenolic substance. By performing potentiostatic electrosynthesis at the suitable electrode potential, it is in fact possible to generate and regenerate two iodinating species, I_2_ or HIO, at the electrode. Considering that iodic acid/iodate is a non-iodinating species and that its formation seems to be almost irreversible, potentiostatic conditions are fundamental to control the generation of iodinating species, which is otherwise impossible in galvanostatic conditions. Once generated, the iodinating species are then able to diffuse back into the bulk of the solution and react with a suitable organic substrate. The organic substrate undergoing this kind of iodination by electrophilic attack of I_2_/HIO must fulfill one condition: its own oxidation peaks must be at higher potentials than that of HIO formation to avoid the oxidation of the substrate. Cyclic voltammetry was also used as an in-process control to monitor the progress of the reaction. Therefore, in order to evaluate the decrease in reactants, the organic species must have an oxidation/reduction peak within the water window and that does not overlap with the KI peaks.

**Figure 3:**
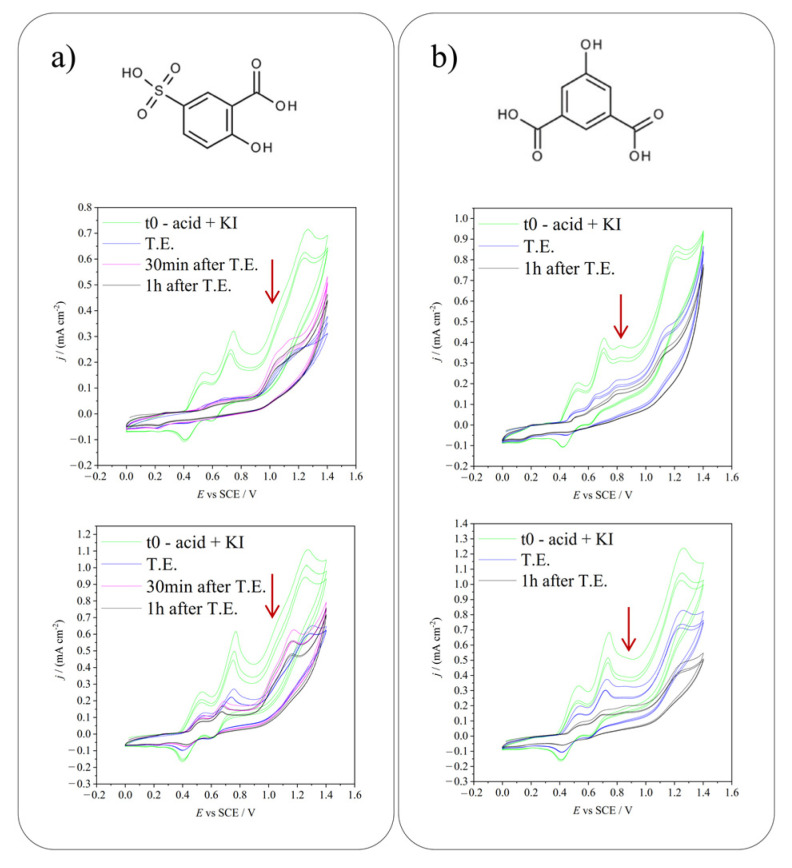
(**a**) 5-sulfosalicylic acid molecule and CVs monitoring the iodination reaction producing HIO (above) and I_2_ (below). (**b**) 5-hydroxyisophthalic acid molecule and CVs monitoring the iodination reaction producing HIO (above) and I_2_ (below). Potentials applied: +0.75 V vs. SCE for HIO production and +0.58 V vs. SCE for I_2_ production. t0 = time 0, before starting the electrolysis, T.E. = theoretical end, calculated considering the quantity of charge needed to convert all the KI in the desired iodinating agent. Scan rate: 20 mV s^−1^. Red arrows indicate the organic reagents oxidation peaks (sulfosalicylic acid in (**a**) and 5-hydroxyisophthalic acid in (**b**)).

After a screening of possible suitable molecules (see Supplementary Materials S2), 5-sulfosalicylic acid and 5-hydroxyisophthalic acid were chosen as molecules for in situ electrochemical iodination. Different experiments were performed to test the best conditions for their iodination: both electro-generation of HIO and I_2_ were evaluated. A neutral environment was used to both favor the iodination (during which protons are released) and to work in conditions in which the generation of the two iodinating species is differentiable. Figure 3 shows the molecules’ structure and the CVs used to monitor the reaction progress. As shown, before starting the electrolysis, in both cases, the CVs show the characteristic three peaks of KI and an additional oxidation one, higher in potential than the second one (a shoulder to the third peak in the 5-sulfosalicylic acid case). This irreversible peak is associated with the oxidation of hydroxyl groups of the organic molecules.

The comparison of pre- and post-electrolysis CVs shows that, both in the case of HIO and I_2_ production, the voltammetric peaks of the reagents decreased with time, confirming that iodination took place. In particular, the reaction seems almost completed at the theoretical end (1 × 10^−4^ Faradays calculated considering the current needed to convert all the KI into the desired iodinating agent, I_2_ or HIO) only in the case of HIO electrosynthesis. The peaks’ decrease continues beyond the theoretical end when generating iodine. This testifies that HIO is a more reactive iodinating agent, that leads to a quicker iodination.

**Figure 4:**
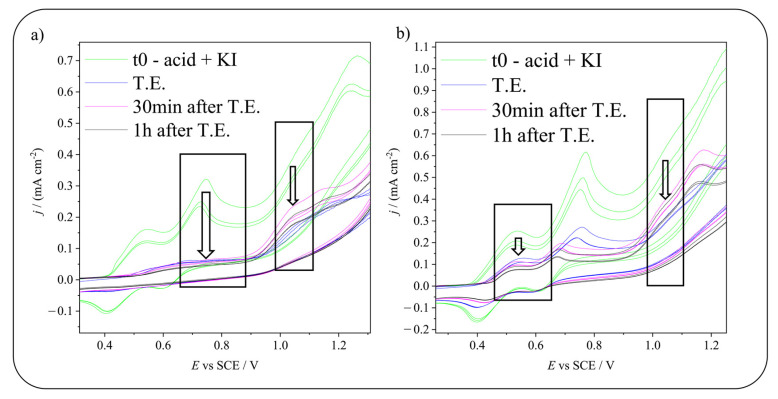
Focus on the current drop obtained during electrolysis producing (**a**) HIO or (**b**) I_2_ for 5-sulfosalicylic acid iodination. In both images, the left arrow indicates the decrease in the peak of the iodinating agent, while the right arrow highlights the diminution of the reagent peak. t0 = time 0, before starting the electrolysis, T.E. = theoretical end, calculated considering the quantity of charge needed to convert all the KI in the desired iodinating agent. Scan rate: 20 mV s^−1^.

**Figure 5:**
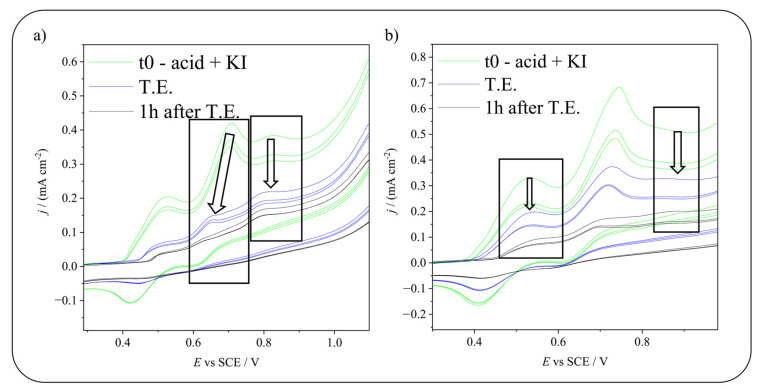
Focus on the current drop obtained during electrolysis producing (**a**) HIO or (**b**) I_2_ for 5-hydroxyisophthalic acid iodination. In both images, the left arrow indicates the decrease in the peak of the iodinating agent, while the right arrow highlights the diminution of the reagent peak. t0 = time 0, before starting the electrolysis, T.E. = theoretical end, calculated considering the quantity of charge needed to convert all the KI in the desired iodinating agent. Scan rate: 20 mV s^−1^.

Figure 4 shows the current drop of the involved peaks during electrolysis for the iodination of 5-sulfosalicylic acid. As can be seen, especially in the case of HIO, by comparing its current drop and the current drop of the substrate, the HIO peak seems to drop faster. This can be due to the fact that some of it does not react with the molecule, but instead, it undergoes disproportionation reactions that consume it. Disproportionation reactions involving HIO are those reported in Equations (1) and (2). In the case of 5-hydroxyisophthalic acid iodination (see Figure 5), from a qualitative point of view, it seems that the reaction involving HIO as an iodinating agent produces instead a faster drop in 5-hydroxyisophthalic acid peak current.

To have a more detailed comprehension of the different reactivity of the two iodinated species, in the case of 5-hydroxyisophthalic acid iodination, a semi-quantitative evaluation on the drop of 5-hydroxyisophthalic acid peak current was performed (see Supplementary Materials S3). The difference between the starting current and the current at the theoretical end is considered to be proportional to the amount of reagent that has been converted into an iodinated product. The current drop is about 44% at the theoretical end in the case of HIO generation and about 35% in that of iodine production, thus confirming that using hypoiodous acid as an iodinating agent leads to higher conversion.

### 2.3. Electrochemical Iodination of 5-Hydroxyisophthalic Acid—Product Analysis

In order to be able to analyze the reaction products, more concentrated electrosynthesis was carried out for 5-hydroxyisophthalic acid. Considering that the possible products are the mono-, di-, and tri-iodinated substrates, electrosynthesis was performed in the presence of a KI excess (4:1). The potential corresponding to the HIO generation was applied (+0.7 V vs. SCE) and the reaction was stopped at almost the theoretical end. The theoretical end was calculated on the basis of the quantity of charge needed to convert all the KI present in the solution in the iodinating agent (1.20 × 10^−2^ Faradays needed, 1.05 × 10^−2^ Faradays applied). UPLC-PDA-HR-MS analysis of the final mixture was then carried out.

**Figure 6:**
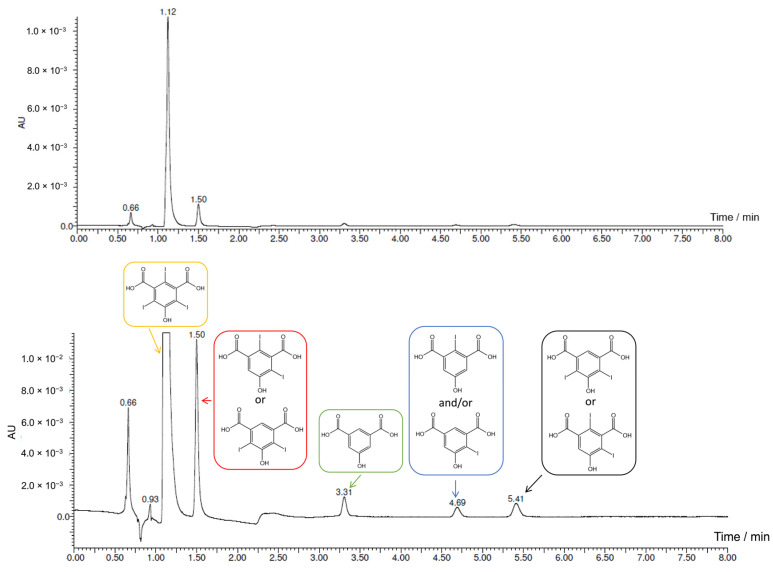
UV chromatograms (λ = 254 nm, isocratic separation: 10% of eluent B at 0.350 mL/min) of the UPLC-PDA-HR-MS analysis. Below is a focus on the region of interest. Peaks identification: tri-iodinated product (1.12 min), di-iodinated products (1.50 and 5.41 min), mono-iodinated product (4.69 min), and 5-hydroxyisophthalic acid (3.31 min).

**Figure 7:**
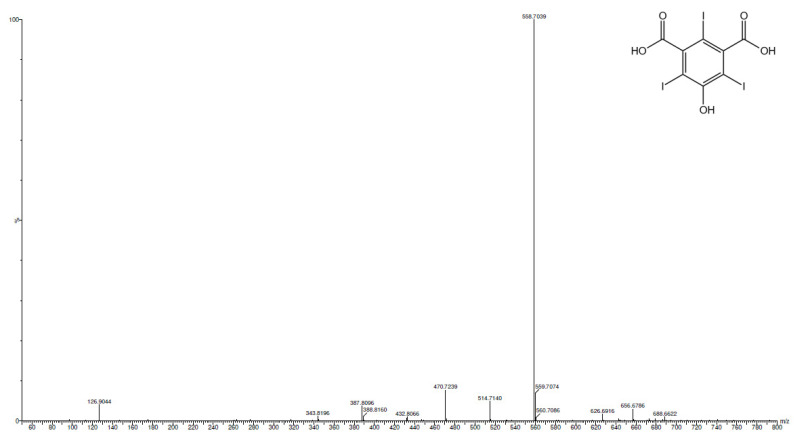

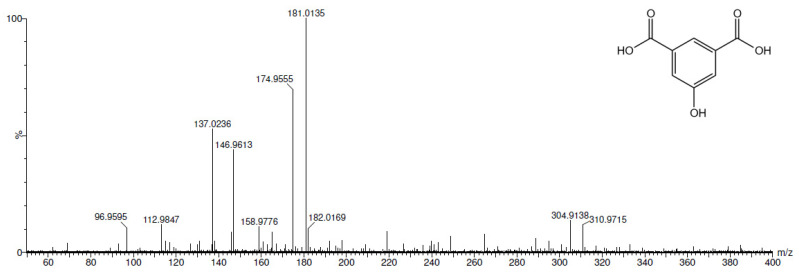
MS analysis of peaks at 1.12 (**above**) and 3.31 (**below**) minutes, corresponding to the tri-iodinated product and the reagent, respectively.

As shown in Figure 6 and Figure 7, the analysis confirmed the almost complete conversion of the reagent. The main peak at 1.12 min corresponds to the tri-iodinated product (*m*/*z* 558.70), while the residual reagent (*m*/*z* 181.01) is the small peak at 3.31 min. Even though it is not possible to evaluate the molar absorption coefficient (ε) due to the commercial unavailability of the products standards, it is reasonable to hypothesize that the reaction almost reached completion and that the tri-iodinated compound is the main product. UPLC-PDA-HR-MS also allowed us to identify three reaction intermediates: the mono-iodinated (4.69 min, *m*/*z* 306.91) and two di-iodinated (1.50 and 5.41 min, *m*/*z* 432.81) compounds. The MS analysis of these peaks are reported in Supplementary Materials S4. Considering the presence of one mono-iodinated and two di-iodinated products, the first iodination probably occurs in the ortho position with respect to the –OH functional group. Both ortho and para positions would be suitable for the aromatic electrophilic substitution, but the former is also favored in terms of steric hindrance. The second iodination does not seem to occur in a preferential position.

### 2.4. Electrochemical Iodination of 5-Sulfosalicylic Acid—Product Analysis

**Figure 8:**
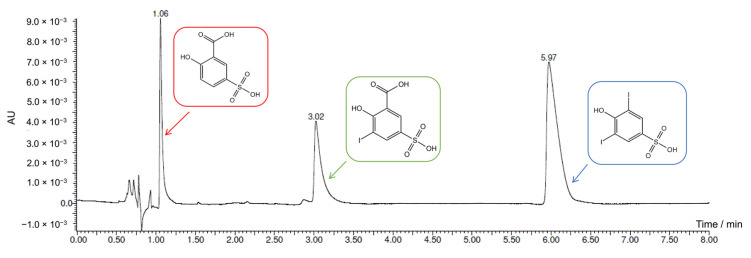
UV chromatograms (λ = 254 nm, isocratic separation: 10% of eluent B at 0.350 mL/min) of the UPLC-PDA-HR-MS analysis. Peaks identification: 5-sulfosalicylic acid (1.06 min), mono-iodinated product (3.02 min), and decarboxylated di-iodinated product (5.97 min).

More concentrated electrosynthesis was also performed for 5-sulfosalicylic acid iodination. Experimental conditions were the same as for the 5-hydroxyisophthalic acid iodination mentioned above. Once again, the theoretical end was calculated on the basis of the quantity of charge needed to convert all the KI present in the solution in the iodinating agent (1.2 × 10^−2^ Faradays needed, 1.0 × 10^−2^ Faradays applied). UPLC-PDA-HR-MS analysis of the final mixture was then carried out, revealing two main products, as shown in Figure 8. MS analyses are reported in the Supplementary Materials S5.

The first peak was identified as the residual reagent, 5-sulfosalicylic acid (*m*/*z* 216.98). The peak at 3.02 min corresponded to the mono-iodinated product (*m*/*z* 342.88), probably the 3-iodo-5-sulfosalicylic acid considering the activation of that position induced by the hydroxyl group. The last peak (5.97 min) had a mass corresponding to the decarboxylated di-iodinated product (*m*/*z* 424.78).

**Figure 9:**
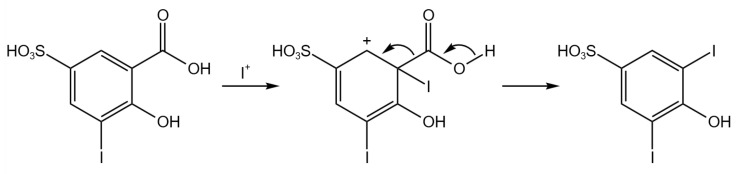
Reaction scheme of the 3,5-diiodo-4-hydroxybenzenesulfonic acid formation.

Its presence could be explained as a fragmentation of the di-iodinated product induced by the MS analysis, but this hypothesis has been discarded considering that no di-iodinated product residual was detected. This peak was then assigned to the 3,5-diiodo-4-hydroxybenzenesulfonic acid, probably formed through a second electrophilic substitution of the “I^+^” electrochemically generated followed by decarboxylation, as shown in Figure 9. This reaction has been already reported in the literature for conventional chemical halogenation [27,28].

3,5-diiodo-4-hydroxybenzenesulfonic acid was separated through reverse phase column chromatography (LiChroprep^®^ RP-18 40–63 µm) using the same eluent as the UPLC-PDA-HR-MS analysis and its structure was confirmed with NMR analysis. The ^1^H, APT (Attached Proton Test), HSQC (Heteronuclear Single Quantum Correlation), and HMBC (Heteronuclear Multiple Bond Correlation) NMR spectra reported in Supplementary Materials S6 confirmed the proposed structure.

## 3. Materials and Methods

### 3.1. Chemicals

All reagents and solvents were of analytical grade (>95%) and commercially available. Phosphoric acid, purchased from Sigma Aldrich, is the only exception (85%). Potassium iodide, sodium phosphate dibasic, potassium nitrate, 5-hydroxyisophthalic acid, 5-aminoisophthalic acid, 3-hydroxybenzyl alcohol, and resorcinol were also purchased from Sigma Aldrich. Iodine resublimed, sodium dihydrogen phosphate dihydrate, and 4-nitrophenol were purchased from Merck. Tri-sodium phosphate dodecahydrate and 5-sulfosalicylic acid were obtained from BDH, while benzoic acid was obtained from Astrochimica. Water, formic acid, and acetonitrile used for UPLC-PDA-HR-MS analysis were MS-grade and were provided by CARLO ERBA Reagents.

### 3.2. Electrochemistry

The study of the electrochemical oxidation of iodide and iodine in aqueous solvent was carried out using cyclic voltammetry. The cell was a three electrode system with a platinum foil counter electrode (CE), GC as working electrodes (WE), and a saturated calomel electrode (SCE) as reference (RE, E_SCE_ = 0.244 V vs. SHE). The cell was also equipped with a bubbler to flux nitrogen and eliminate the dissolved oxygen in the solution. All the solutions were degassed for about 10 min before performing the analysis. The dependency of peak potential on pH was studied using phosphate buffer 0.5 M at various pHs. The buffers were prepared using the suitable phosphate salts and the pH was measured through pH-meter. The solutions used had pH 1.9, 6.5, 7.0, and 10.7. Analyte concentrations were 1 and 2 mM.

The experimental setup for the electrolysis of KI in the presence of a phenolic substance for the voltammetric study was the same, but no nitrogen was fluxed in this case. The working electrode was a carbon cloth AvCarb^®^ 1209HCB (AvCarb Material Solution, Lowell, MA, USA) for the electrolysis and GC for the monitoring CVs. The reaction solution was maintained under stirring throughout the electrolysis duration. Electrolyte for electrosynthesis experiments was a buffer phosphate solution 0.5 M pH 6–7. The concentration of both organic substrates and KI in the electrolysis test was 1 mM for the voltammetric study. The concentration of organic substrates screened before was 1 mM.

More concentrated electrosynthesis for UPLC-PDA-HR-MS (Ultra Performance Liquid Chromatography—PhotoDiode Array—High Resolution—Mass Spectroscopy) analysis was carried out using organic molecule 15 mM and KI 60 mM. A cell divided by a cation exchange membrane Nafion^®^ 117 (DuPont, Wilmington, DE, USA) was used in this case. The anode and cathode were a carbon cloth AvCarb^®^ 1209HCB (AvCarb Material Solution, Lowell, MA, USA) and a platinum foil, respectively (see Supplementary Materials S7 for the set-up scheme and picture). The reference electrode (SCE) was equipped with a KNO_3_ 1 M double salt bridge. The anolyte and catholyte (both 100 mL) were potassium phosphate buffer 0.5 M pH 6.

Both cyclic voltammetries and chronoamperometries were performed using the Autolab potentiostat/galvanostat PGSTAT204 by Metrohm.

### 3.3. UPLC-PDA-HR-MS and NMR

The UPLC-PDA-HR-MS analyses were performed on an Acquity UPLC I Class (Waters, Milford, MA, USA). The column was an Acquity UPLC HSS T3 (100 × 2.1 mm, 1.8 μm) fitted with a VanGuard cartridge (Waters). The column temperature was 30 °C and the eluents were: A, water + 0.1% formic acid and B, acetonitrile + 0.1% formic acid. Isocratic separation: 10% of eluent B at 0.350 mL/min. Injection volume: 2 µL of 250 μM solution. The detector was an Acquity UPLC PDA Detector and the wavelength selected for the analysis was 254 nm. A Synapt G2-Si QTof (Waters) High Resolution mass spectrometer equipped with a Zspray ESI-probe was used in ESI negative ionization mode. Optimized source parameters: capillary −2.0 kV, cone 40, source temperature 120 °C, desolvatation temperature 150 °C, desolvatation gas flow rate 600 L/h, and full scan range 50–800 *m*/*z*. The lock mass compound was leucine enkephalin. The software was MassLynx^TM^ v4.2 software (Waters).

NMR spectra were performed using an Avance NEO 400 spectrometer (Bruker, Billerica, MA, USA) equipped with a “BBI 400 MHz S1” probe with Z gradient.

## 4. Conclusions

In sum, the electrochemical iodination of two molecules, 5-sulfosalicylic acid and 5-hydroxyisophthalic acid, is reported in this paper and seems to represent an effective and promising way to produce iodinated compounds using a cheap, simple, and green strategy as a valid alternative to traditional synthetic methods. The electrochemical behavior of iodide and iodine in water on carbonaceous anodes was studied at a preliminary stage to understand which species were involved during the electrochemical oxidation. The CVs revealed that both iodide and iodine gave rise to three anodic peaks on GC, where two iodinating species, I_2_ and HIO, were generated at the first and second peaks, respectively. The potentials for their generation were selected as the most suitable for iodination reactions. A screening of organic molecules susceptible to electrophilic attack for in situ electrochemical iodination was performed through voltammetric studies and 5-sulfosalicylic acid and 5-hydroxyisophthalic acid were selected. Potentiostatic electrolysis in the presence of both the molecule and KI were conducted and CVs were successfully used as a monitoring technique for their iodination. The iodinating power of I_2_ and HIO species were compared and quicker iodination was observed with the latter, which appeared more reactive. UPLC-PDA-HR-MS analysis on more concentrated electrolysis for the iodination of both molecules revealed an almost complete conversion of the reagent with the formation of iodinated products.

In conclusion, this work successfully investigated the electrochemical iodination of two molecules. However, it is necessary to expand the case history so that a true synthetic method can be defined. The results of this study will hopefully provide the base for future studies and improvements, leading to even greener and more versatile protocols.

## Data Availability

Not applicable.

## Supplementary Materials

The following supporting information can be downloaded at: https://www.mdpi.com/article/10.3390/molecules28145555/s1, Supplementary Materials S1: Data for the identification of 2A and 3A reactions. Supplementary Materials S2: Screening of suitable phenolic molecules susceptible of electrophilic attack. Supplementary Materials S3: Electrosynthesis current drop in the case of 5-hydroxyisophthalic acid. Supplementary Materials S4: 5-hydroxyisophthalic acid iodination mixture MS analyses. Supplementary Materials S5: 5-sulfosalicylic acid iodination mixture MS analyses. Supplementary Materials S6: NMR spectra of 3,5-diiodo-4-hydroxybenzenesulfonic acid. Supplementary Materials S7: Experimental set-up for electroiodination.
