# Supplementary material for: Electrochemical Iodination through the In Situ Generation of Iodinating Agents: A Promising Green Approach
Source: Molecules. 2023 Jul 20;28(14):5555. doi: 10.3390/molecules28145555 (PMC10383702; doi:10.3390/molecules28145555)
Supplement: Supplementary file 1 [file molecules-28-05555-s001.zip › molecules-2439611-supplementary.pdf]

Electrochemical Iodination through the In Situ Generation of Iodinating Agents: A Promising Green Approach

Letizia Sorti [1], Fiammetta Vitulano [1], Elia Cappellini [1], Fulvio Uggeri [2], Carlo Francesco Morelli [1], Guido Sello [1], Alessandro Minguzzi [1,3]*, Alberto Vertova [1,3]

Adresses

[1] Laboratory of Applied Electrochemistry, Dipartimento di Chimica, Università degli Studi di Milano, via Golgi, 19, 20133, Milano, Italy

[2] Bracco SpA, via Caduti di Marcinelle, 13, 20134, Milano, Italy

[3] Consorzio Interuniversitario Nazionale per la Scienza e Tecnologia dei Materiali – INSTM. Via G. Giusti 9, 50121, Firenze, Italy

***Corresponding Author alessandro.minguzzi@unimi.it**

Supplementary Materials:

S1: Data for the identification of 2A and 3A reactions.

List of the possible reaction to which anodic peaks 2A and 3A could be assigned.

2A	$I_2 + 2\ H_2O \rightarrow 2HIO + 2H^+ + 2e^-$	$E_{vs\ SHE}\ (V) = 1.354 - 0.0591\ pH$	(S1)
	$I^- + H_2O \rightarrow HIO + H^+ + 2e^-$	$E_{vs\ SHE}\ (V) = 0.987 - 0.0295\ pH$	(S2)
	$I_3^- + 3\ H_2O \rightarrow 3HIO + 3H^+ + 4\ e^-$	$E_{vs\ SHE}\ (V) = 1.213 - 0.0443\ pH$	(S3)
3A	$HIO + 2\ H_2O \rightarrow IO_3^- + 5\ H^+ + 4\ e^-$	$E_{vs\ SHE}\ (V) = 1.134 - 0.0739\ pH$	(S4)
	$IO^- + 2\ H_2O \rightarrow IO_3^- + 4\ H^+ + 4\ e^-$	$E_{vs\ SHE}\ (V) = 0.972 - 0.0591\ pH$	(S5)
	$I_3^- + 9\ H_2O \rightarrow 3\ HIO_3 + 15\ H^+ + 16\ e^-$	$E_{vs\ SHE}\ (V) = 1.145 - 0.0554\ pH$	(S6)
	$I_3^- + 9\ H_2O \rightarrow 3\ IO_3^- + 18\ H^+ + 16\ e^-$	$E_{vs\ SHE}\ (V) = 1.154 - 0.0665\ pH$	(S7)
	$I_2 + 6\ H_2O \rightarrow 2\ HIO_3 + 10\ H^+ + 10\ e^-$	$E_{vs\ SHE}\ (V) = 1.169 - 0.0591\ pH$	(S8)
	$I_2 + 6\ H_2O \rightarrow 2\ IO_3^- + 12\ H^+ + 10\ e^-$	$E_{vs\ SHE}\ (V) = 1.178 - 0.0709\ pH$	(S9)
	$I^- + 3\ H_2O \rightarrow HIO_3 + 5\ H^+ + 6\ e^-$	$E_{vs\ SHE}\ (V) = 1.077 - 0.0493\ pH$	(S10)
	$I^- + 3\ H_2O \rightarrow IO_3^- + 6\ H^+ + 6\ e^-$	$E_{vs\ SHE}\ (V) = 1.085 - 0.0591\ pH$	(S11)

Figure S1 and **Table S1** reporting the experimental linear dependencies on pH of first, second and third oxidation peak reported in **Figure 2a)** of KI 2 mM on a GC electrode.

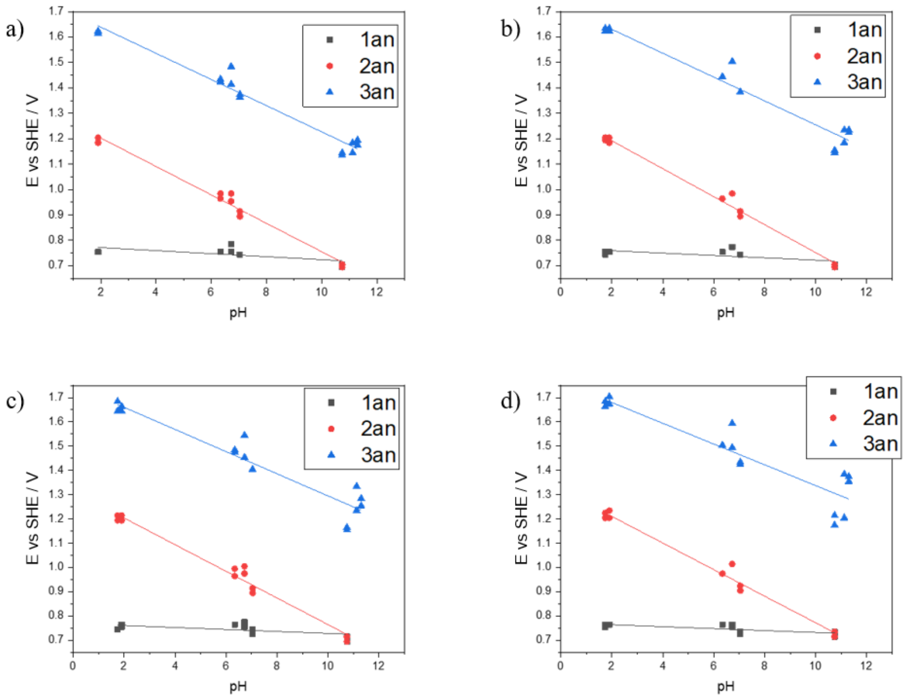

Figure S1: *Plots potential (E) vs pH of anodic peak at a)10, b) 20, c) 50 and d) 100 mV s*$^{-1}$*. WE = GC, RE = SCE, CE = Pt, electrolyte = phosphate buffer solution 0.5 M at various pH, analyte = KI 2 mM.*

Table S1: *Values of intercepts and slopes obtained from plot E vs pH for each oxidation peak at each scan rate.*

	**10 mV/s**			**20 mV/s**	
**Peak**	**Intercept (vs SHE)**	**Slope**	**Peak**	**Intercept (vs SHE)**	**Slope**
**1A**	0.78 ± 0.02	-0.006 ± 0.002	**1A**	0.76 ± 0.01	-0.005 ± 0.002
**2A**	1.31 ± 0.02	-0.056 ± 0.003	**2A**	1.30 ± 0.01	-0.055 ± 0.002
**3A**	1.74 ± 0.03	-0.051 ± 0.003	**3A**	1.72 ± 0.03	-0.047 ± 0.003
	**50 mV/s**			**100 mV/s**	
**Peak**	**Intercept (vs SHE)**	**Slope**	**Peak**	**Intercept (vs SHE)**	**Slope**
**1A**	0.77 ± 0.01	-0.004 ± 0.002	**1A**	0.77 ± 0.01	-0.004 ± 0.001
**2A**	1.31 ± 0.02	-0.055 ± 0.003	**2A**	1.32 ± 0.02	-0.055 ± 0.003
**3A**	1.75 ± 0.03	-0.046 ± 0.004	**3A**	1.77 ± 0.04	-0.043 ± 0.005

S2: Screening of suitable phenolic molecules susceptible of electrophilic attack.

The potential screening of suitable phenolic molecules susceptible of electrophilic attack is reported in **Table S2**.

Table S2: *Electrophilic attack susceptible phenolic substances screening in phosphate buffer 0.5 M pH 6.5.*

Reactant	Oxidation peak potential vs SCE (V)	Observation (pH 6.5)
**Phenol**	-	Direct oxidation, polymerization products
**4-nitrophenol**	~ 0.89	Probable polymerization
**Resorcinol**	~ 0.7	Potential not high enough
**5-sulfosalicylic acid**	~ 0.9	Can be used
**Benzoic acid**	~ 0.93	Very little, overlapped with iodate peak
**5-aminoisophthalic acid**	~ 0.82	Can be used
**5-hydroxyisophthalic acid**	~ 0.83	Can be used
**3-hydroxybenzyl alcohol**	~ 0.65	Potential not high enough

S3: Electrosynthesis current drop in the case of 5-hydroxyisophthalic acid.

Average current drop of 5-hydroxyisophthalic acid in the case of HIO (**Table S3**) and I_2 (**Table S4**) production are below reported.

Table S3: *Report of peak current values and current drop of 5-hydroxyisophthalic acid peak for different iodination (iodinating agent: HIO) at the start and at the theoretical end of the reaction.*

HIO Sample	t_0 peak current (j / mA cm^{-2})	TE peak current (j / mA cm^{-2})	Current drop (%)
**1**	0.3287	0.1786	45.7
**2**	0.2988	0.1632	45.5
**3**	0.3905	0.2247	42.5
**4**	0.3963	0.2314	41.6
**5**	0.3840	0.2183	43.2

Table S4: *Report of peak current values and current drop of 5-hydroxyisophthalic acid peak for different iodination (iodinating agent: I_2) at the start and at the theoretical end of the reaction.*

I_2 Sample	t_0 peak current (j / mA cm^{-2})	TE peak current (j / mA cm^{-2})	Current drop (%)
**1**	0.5727	0.3822	33.3
**2**	0.5203	0.3276	37.0
**3**	0.3640	0.2390	34.3

S4: 5-hydroxyisophthalic acid iodination mixture MS analyses

MS analyses of peaks at 1.50 (di-iodinated product), 4.69 (mono-iodinated product) and 5.41 (di-iodinated product) minutes are reported in **Figure S2**, **Figure S3** and **Figure S4**.

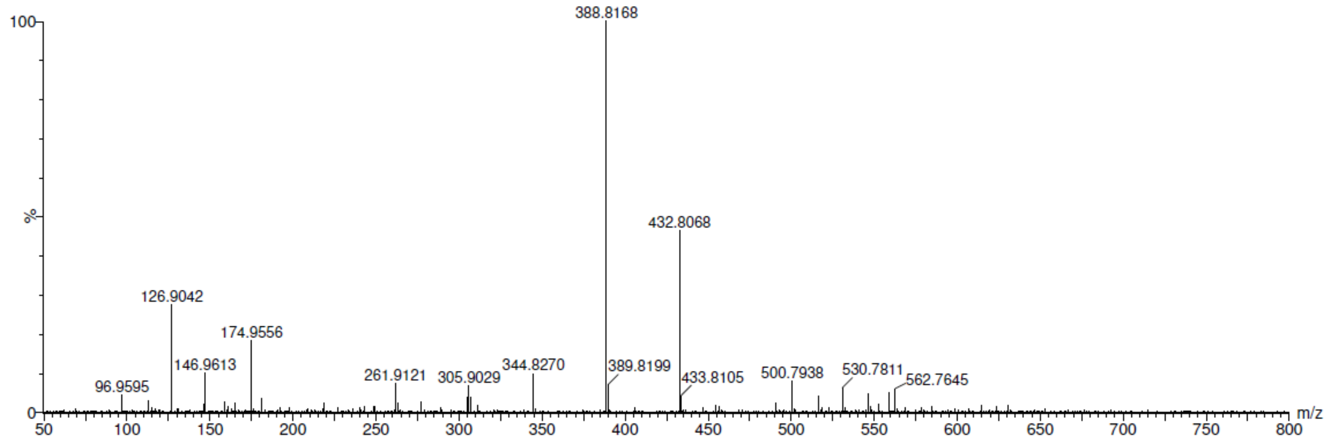

__Figure S2:__ MS analysis of the 1.50 minutes peak, corresponding to the di-iodinated product (m/z 432.81). m/z 388.82 is the resulting product of the loss of a -COO⁻ group.

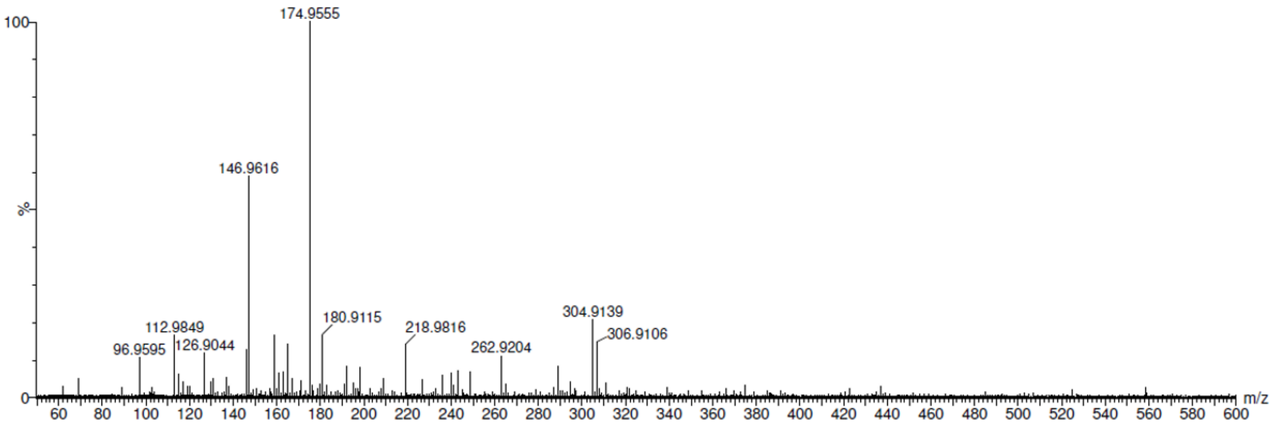

__Figure S3:__ MS analysis of the 4.69 minutes peak, corresponding to the mono-iodinated product (m/z 306.91). The ion with m/z 174.96 is referred to the analytical background (it is also present in the blank).

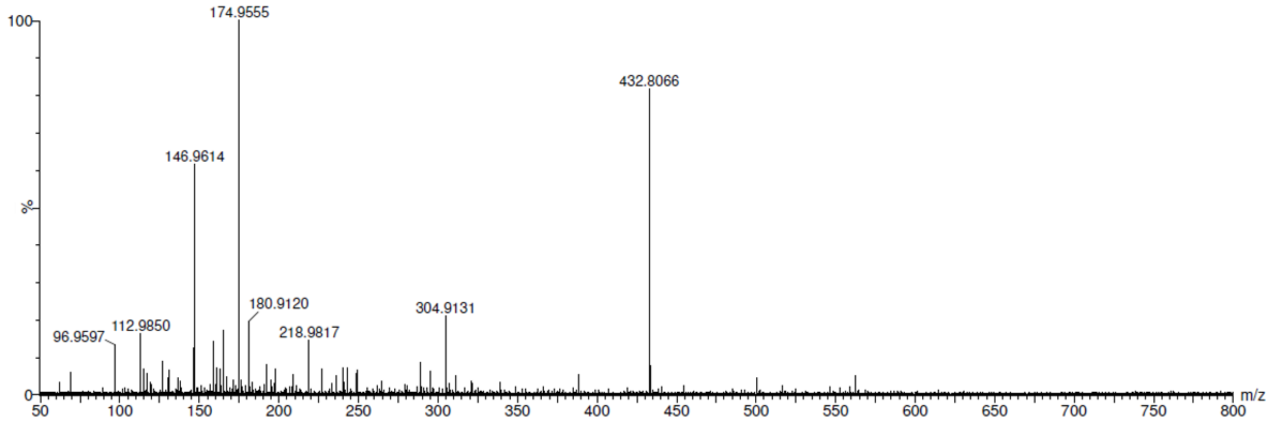

__Figure S4:__ MS analysis of the 5.41 minutes peak, corresponding to the di-iodinated product (m/z 432.81). The ion with m/z 174.96 is referred to the analytical background (it is also present in the blank).

S5: 5-sulfosalicylic acid iodination mixture MS analyses

MS analyses of peaks at 1.06 (5-sulfosalicylic acid), 3.02 (mono-iodinated product) and 5.97 (decarboxylated di-iodinated product) minutes are reported in **Figure S5**, **Figure S6** and **Figure S7**.

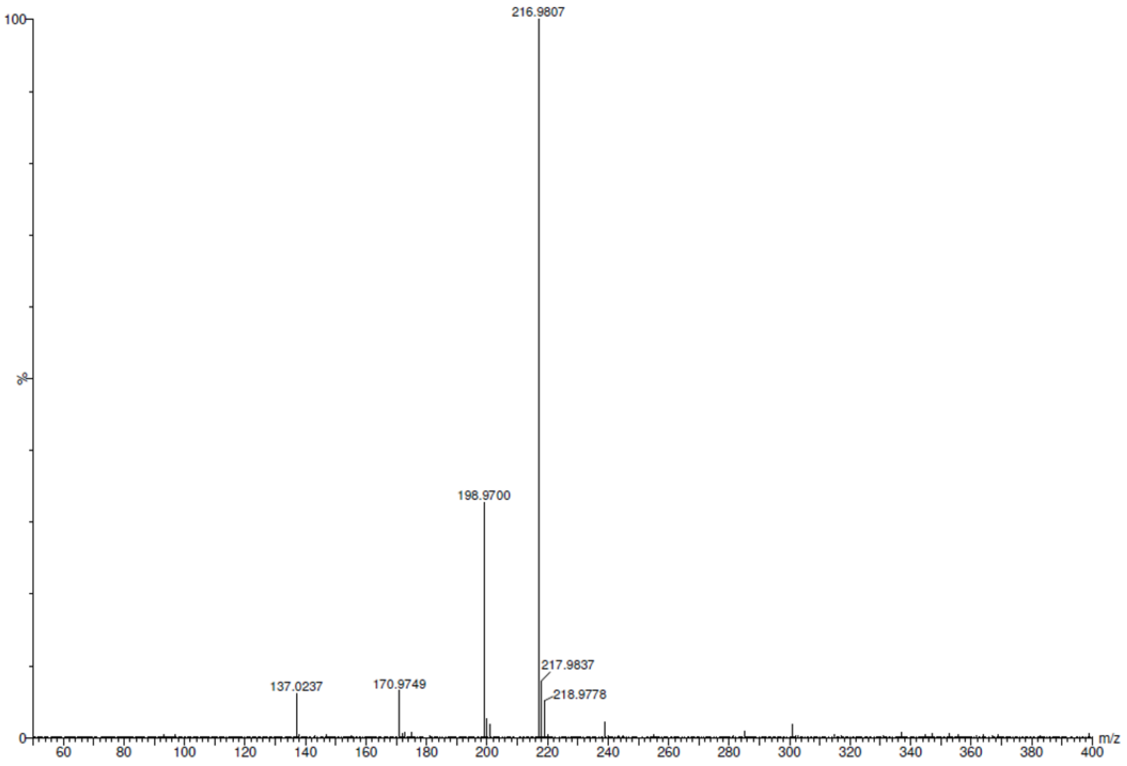

Figure S5: *MS analysis of the 1.06 minutes peak, corresponding to the 5-sulfosalicylic acid (m/z 216.98).*

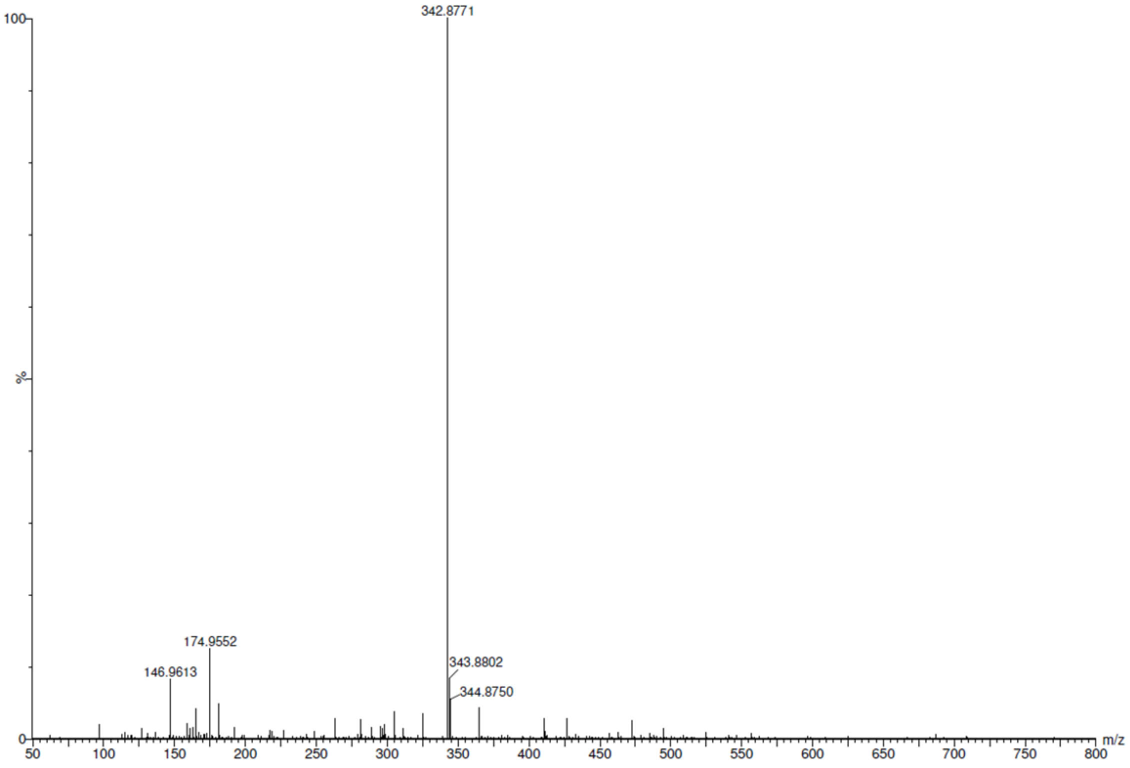

Figure S6: *MS analysis of the 3.02 minutes peak, corresponding to the mono-iodinated product (m/z 342.88).*

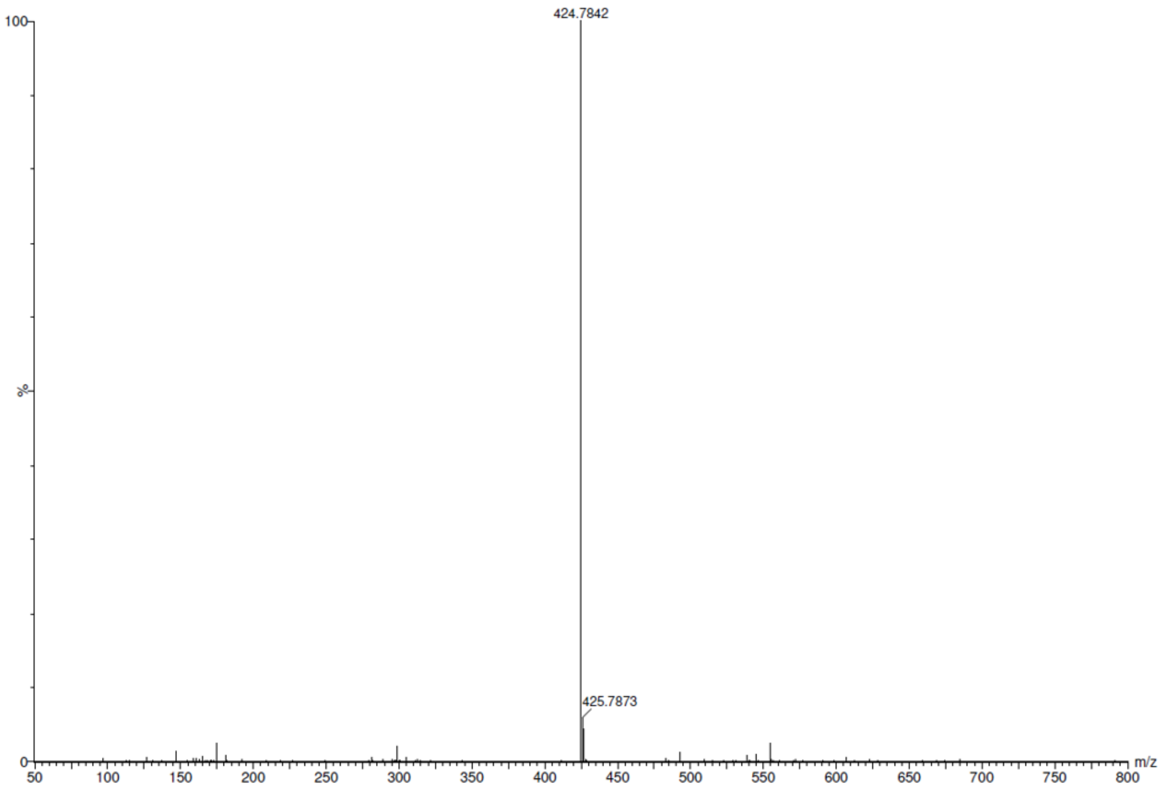

Figure S7: *MS analysis of the 5.97 minutes peak, corresponding to the decarboxylated di-iodinated product (m/z 424.78).*

S6: NMR spectra of 3,5-diiodo-4-hydroxybenzenesulfonic acid

5-sulfosalicylic acid iodination product 3,5-diiodo-4-hydroxybenzenesulfonic acid ^{1}H, APT, HSQC and HMBC NMR spectra are reported in **Figure S8**, **Figure S9**, **Figure S10** and **Figure S11** respectively.

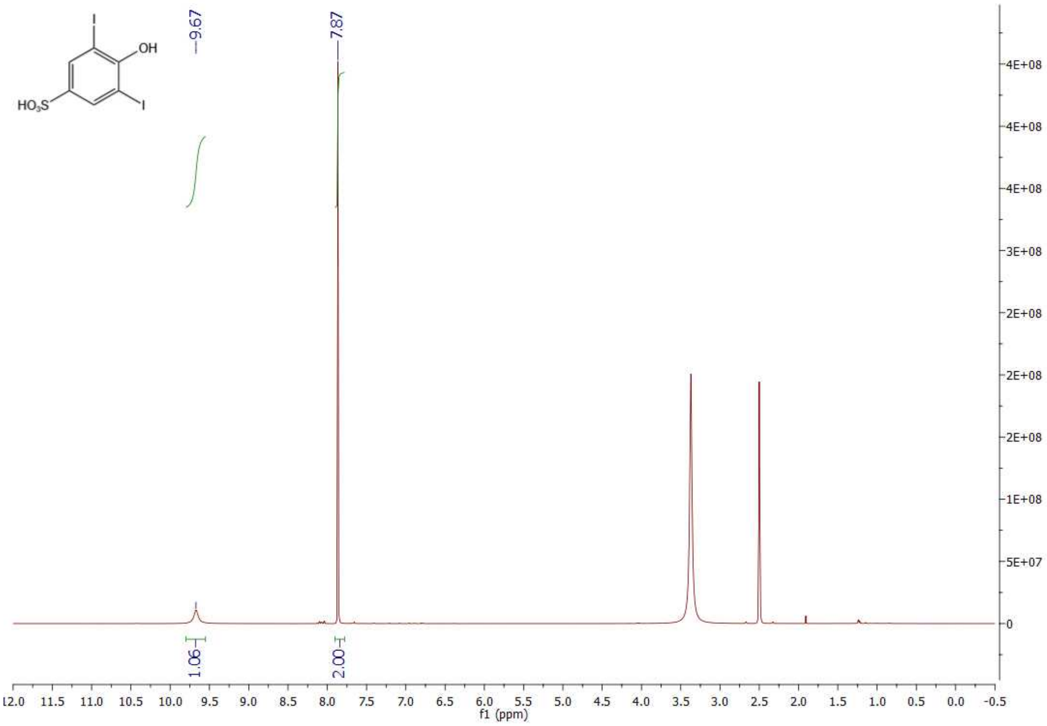

Figure S8: *^{1}H-NMR spectrum of 3,5-diiodo-4-hydroxybenzenesulfonic acid (decarboxylated di-iodinated product, peak at 5.97 min in UPLC-PDA-HR-MS analysis). ^{1}H-NMR (400 MHz, DMSO- d_6) δ 9.67 (s, 1H, OH), 7.87 (s, 2H, H-2 and H-6).*

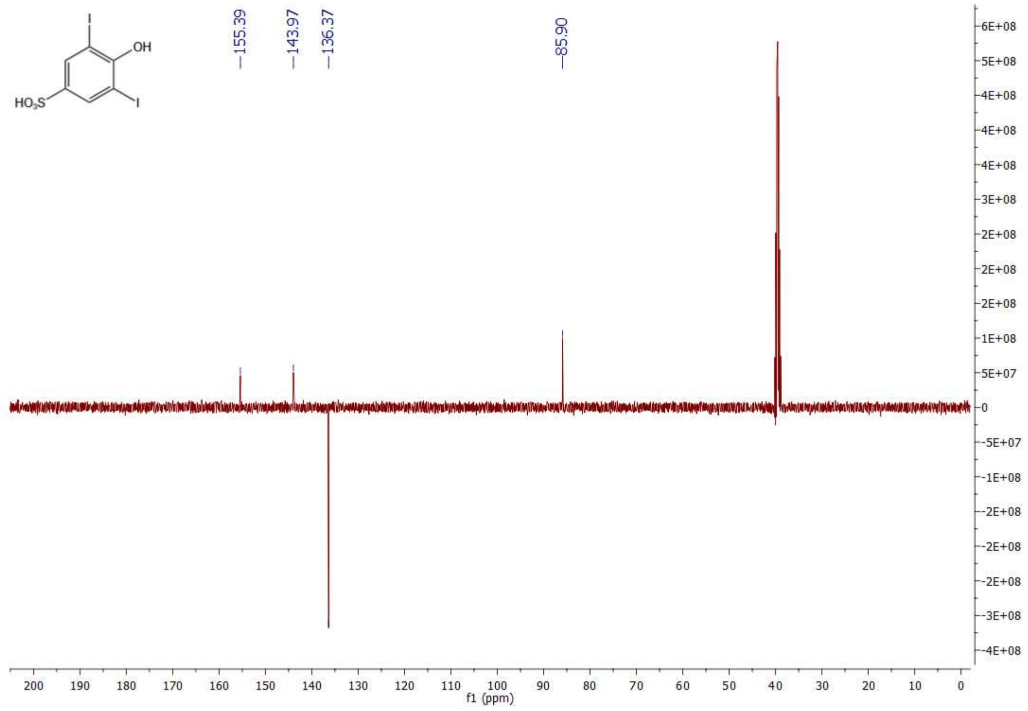

Figure S9: *APT (Attached Proton Test) NMR spectrum of 3,5-diiodo-4-hydroxybenzenesulfonic acid (decarboxylated di-iodinated product, peak at 5.97 min in UPLC-PDA-HR-MS analysis). Positive peaks are secondary and quaternary carbons, negative peaks are primary and tertiary carbons. ^{13}C-NMR (101 MHz, DMSO-d_6) δ 155.39 (C-4), 143.97(C-1), 136.37 (C-2, C-6) and 85.90 (C-3, C-5).*

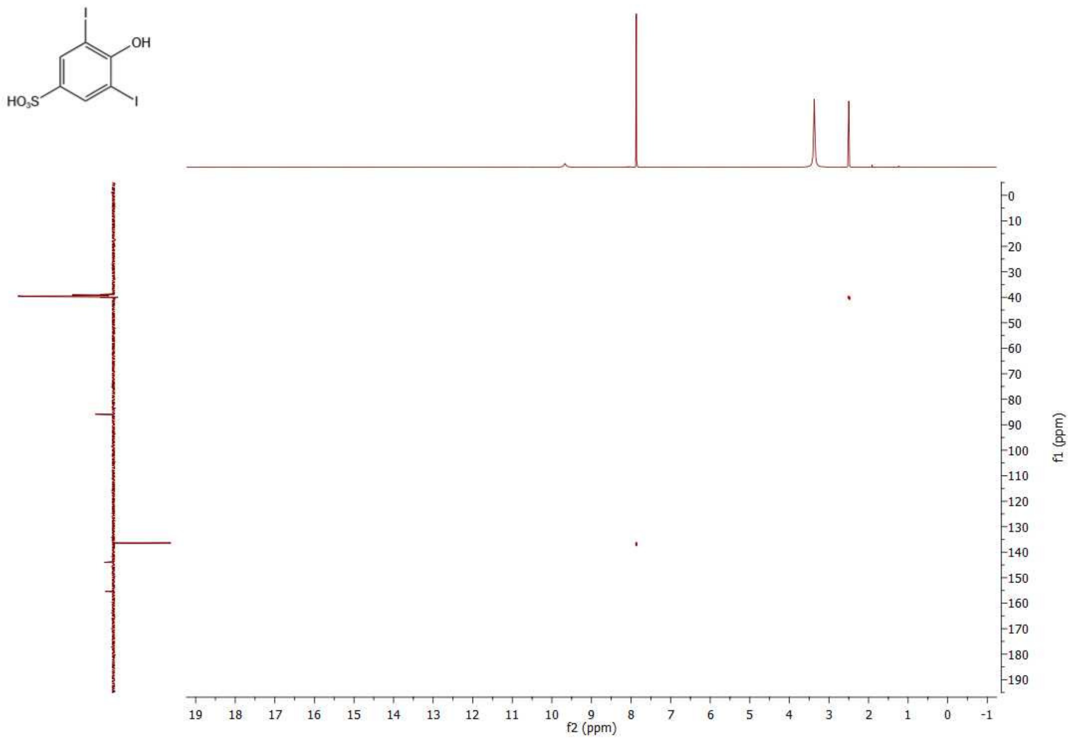

***Figure S10**: HSQC NMR (Heteronuclear Single Quantum Correlation) spectrum of 3,5-diiodo-4-hydroxybenzenesulfonic acid (decarboxylated di-iodinated product, peak at 5.97 min in UPLC-PDA-HR-MS analysis).*

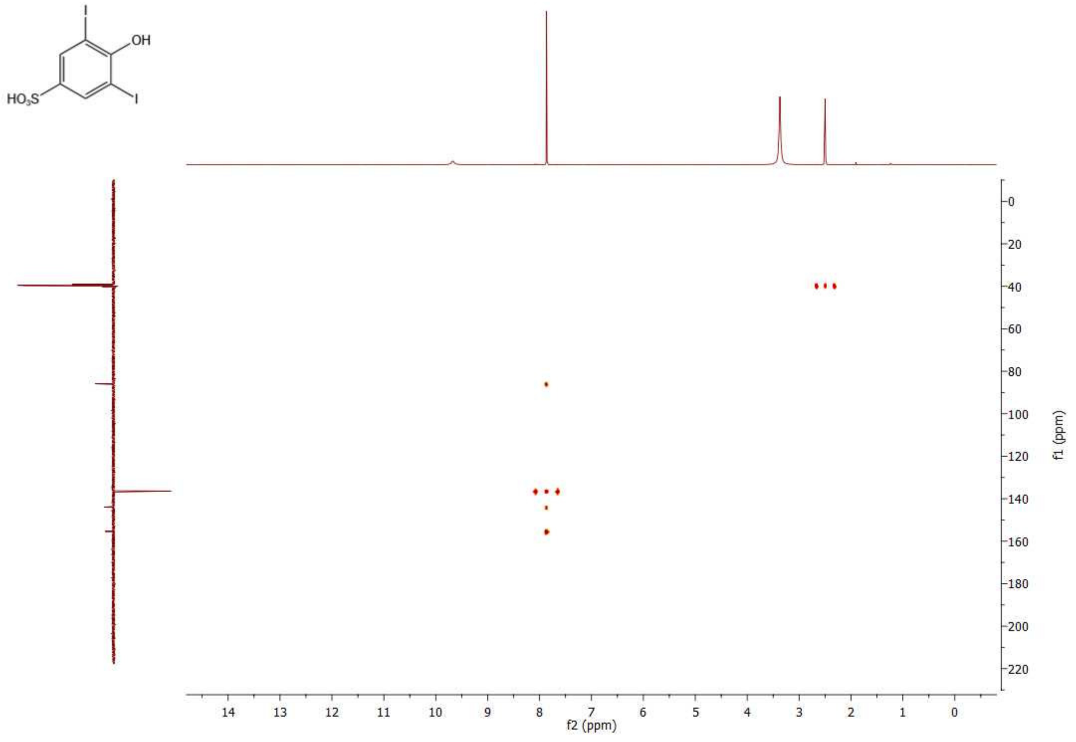

***Figure S11:** HMBC NMR (Heteronulear Multiple Bond Correlation) spectrum of 3,5-diiodo-4-hydroxybenzenesulfonic acid (decarboxylated di-iodinated product, peak at 5.97 min in UPLC-PDA-HR-MS analysis).*

S7: Experimental set-up for electroiodination

Experimental set-up for electroiodination of 5-hydroxyisophthalic acid and 5-sulfosalicylic acid 15 mM.

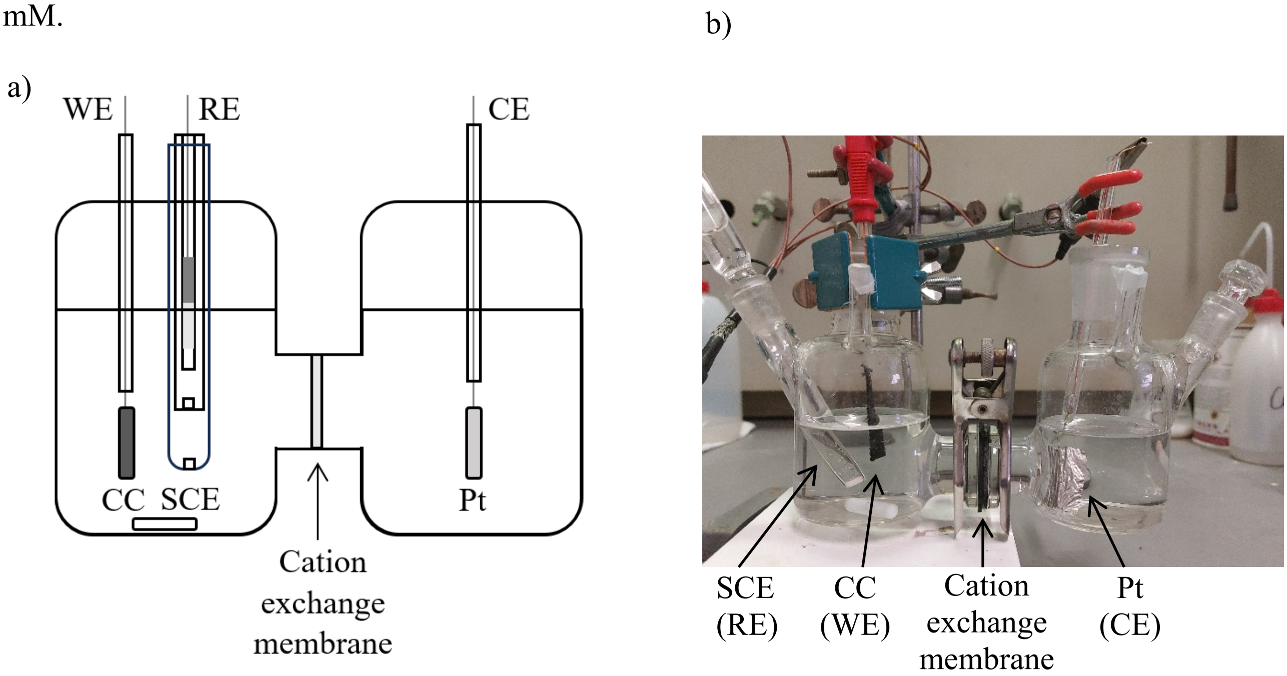

Figure S12: *a) scheme and b) picture of the divided cell for electrosynthesis of 5-hydroxyisophthalic acid and 5-sulfosalicylic acid 15 mM. WE= working electrode, RE= reference electrode (equipped with a* KNO_3 *1 M double salt bridge), CE= counter electrode, CC= carbon cloth, SCE= saturated calomel electrode.*
